# Reaching hard to reach youth: Who are the inactive youth?

**DOI:** 10.1177/13591053261430656

**Published:** 2026-03-23

**Authors:** Reidar Säfvenbom, Jennifer P. Agans, Elin Kolle, Tommy Haugen

**Affiliations:** 1 Norwegian School of Sport Sciences, Oslo, Norway; 2 The Pennsylvania State University, University Park, PA, USA; 3 University of Agder, Kristiansand, Norway

**Keywords:** sedentary behavior, adolescents, determinants, change, physical activity

## Abstract

Despite growing knowledge emphasizing benefits of physical activity, the inactivity rate in youth seems to remain. The present study suggests that one reason for this is lack of knowledge about the inactive and that exploration of differences in every daily life-challenges of inactive and active youth are needed. Applying multivariate analysis of variance and classification and regression tree analysis, differences between the two groups were seen. Results show associations between demographic variables and inactivity, yet when adding psycho-social variables, the accuracy improved significantly. The results suggest that inactive youth seem to live more challenging lives compared to their active peers. When we feed statistical analyses with only demographic data such as age, gender, social class and ethnicity, we may fail to examine the role of important psycho-social variables and thus miss beneficial knowledge about inactive youth and a pedagogical potential for developing appropriate interventions.

## Introduction

Physical activity (PA) is a vital component of a healthy lifestyle, offering various benefits for individuals of all ages (Bull et al., 2020; Warburton and Bredin, 2017). Involvement in PA is particularly pronounced among children and youth and may play a pivotal role in their physical development (Bull et al., 2020; Janssen and Leblanc, 2010), as well as their social and psychological development (Eime et al., 2013; van Den Broek et al., 2024). For example, in addition to biological benefits, participation in PA-based activities is associated with reduced levels of depression and anxiety (Dale et al., 2019; McInerney and Deschênes, 2025; McMahon et al., 2017; Steinsbekk et al., 2026), social inclusion and social identity development (Anderson-Butcher, 2019; Bruner et al., 2017; Säfvenbom et al., 2023), and enhanced thinking, learning, and judgment skills (Donnelly et al., 2016; Marques et al., 2018).

Due to the positive effects of physical activity, public health guidelines recommend that children and adolescents should engage in at least 60 minutes of moderate to vigorous physical activity (MVPA) every day (Bull et al., 2020), but physical inactivity remains a growing problem (Milton et al., 2024). Data from 47,000 young people across 18 European countries shows that only 29% of children and 29% of adolescents meet the recommendation (Steene-Johannessen et al., 2020), and data from Norway shows that whilst almost 90% of 6 year olds meet PA recommendations, only 40%–50% of 15-olds do the same; adolescents spend approximately 70% of their waking hours in sedentary activities.

Evidence also shows that inactive youth tend to become more inactive over time (Steene-Johannessen et al., 2021) and they are often difficult to engage through interventions (Love et al., 2019). Since these youth are challenging to reach and less likely to participate in voluntary PA, such as organized sports, schools have frequently been used as a mandatory setting to target them. However, the limited success of such efforts (van Sluijs et al., 2021) suggests that mandatory participation may not be the most effective or only approach. In fact, requiring participation in PA during the school day may reinforce negative attitudes toward exercise potentially leading to lifelong disengagement (Cuevas-Campos et al., 2020). Therefore, it is important to examine inactive youth more closely to understand their characteristics and why they remain difficult to reach and resistant to interventions.

It is widely recognized that physical inactivity often parallels social inequities, such that disparities in PA align with marginalization related to gender, race/ethnicity, socioeconomic status, newcomer status (Persson and Eriksen, 2025; Spaaij et al., 2014), weight (Bevan et al., 2023), sexual orientation, and gender identity (Denison et al., 2021). However, existing evidence regarding determinants that affect PA behaviors is not convincing. Significant percentages of inactive persons are to be found in all groups and segments of youth in society (van Sluijs et al., 2021), indicating that there are variables beyond gender, socioeconomic status, and ethnicity that hinder involvement in existing physical activity contexts. To increase necessary knowledge in the area and to support more appropriate interventions, such variables need to be identified. The limited understanding of the psychosocial characteristics of these youth may reduce the effectiveness of interventions (Cortis et al., 2017) and potentially lead to unintended consequences. Accordingly, more knowledge could pave the way for programs, pedagogies, and didactics that fit developmental trajectories and needs that support positive experiences and behavior change in these youth. To achieve this, we need a deeper understanding of who these youth are, including whether and how they differ from their more physically active peers. Given the interconnectedness of biological, psychological, and social systems in human development (Overton, 2014), physical activity interventions aiming to change biological systems (i.e. physical fitness), as well as mental, social, and behavioral systems, could benefit by also accounting for factors at the psychosocial and social context levels.

### Theoretical approach

To date, much of the research on adult physical inactivity has centered on personal responsibility (e.g. the Theory of Planned Behavior (Ajzen, 1991); the Transtheoretical Model (Prochaska and Velicer, 1997); the Health Action Process Approach (Schwarzer et al., 2008) or contextual barriers (e.g. leisure constraints; Hubbard and Mannell, 2001), and these theories can also be applied to youth. However, studies anchored in more dynamic and process-relational paradigms (Lerner, 2018; Overton, 2014), acknowledging relational analysis of interactions between physiological, emotional, cognitive, behavioral, and other systems within youth, as well as the interaction between youth and various contexts based on PA, have been shown to add valuable knowledge (Erdvik, 2020; Säfvenbom et al., 2023).

Within these paradigms, relational developmental systems (RDS) theories are considered progressive (Lerner, 2018) regarding social equality in health and education; RDS-based research seeks not only to describe or explain human development and behavior, but also to optimize developmental processes (Lerner, 2018). According to RDS, humans are socially embedded in a certain time of history, and they are 100% physical and 100% mental, which means that determinants of behavior must be understood as interrelated with each other rather than being additive or multiplicative (Lerner, 2018). This indicates dynamic and relational perspectives and analyses and means that when developing activity programs for youth we need to identify variances in “goodness of fit” between personal characteristics (e.g. biological, mental, social, behavioral) of the young person and current contextual specificities (e.g. physical activity interventions). While a high goodness of fit reflects alignment between needs and characteristics of the youth and content, logics, meanings, and values provided through the context, a low goodness of fit reflects the opposite (Agans et al., 2013). Unfortunately, this perspective has been applied to PA more in theory (Agans, 2024; Agans et al., 2013; Erdvik, 2020; Säfvenbom et al., 2023) than it has been applied as a framework for interventions or action research programs.

### The present study

From an RDS-perspective as presented above there is a need to understand who the inactive youth are and how inactive youth meet PA contexts. To understand inactive youth requires exploration of factors beyond the typically-studied variables such as gender, socio-economic status, and ethnicity to examine whether disparity in these variables is associated with variance in a relational goodness of fit between youth and physical activity interventions.

With most of the literature on physical inactivity focusing on demographic disparities, the present study represents an exploratory initial step toward better understanding the psychosocial characteristics and social contexts of inactive youth. As such, we analyzed existing data from a study of PA, PE, and sport behavior among youth in Norway. In keeping with the RDS approach framing this work, we examined a diverse set of variables representing different levels of the developmental system (i.e. eagerness for PA, gender, perceived socio-economic status, best friends’ values and behavior, quality of life, perceived competences). However, because these variables represent the psychosocial and social context factors measured in the utilized data set, rather than an examination of an a priori set of hypotheses, the study is considered exploratory. The aim of the study is therefore to explore differences between physically active and physically inactive young people, applying demographic variables such as gender, perceived socioeconomic status, and newcomer status before adding psychosocial variables such as perceived competences, personal growth, and quality of life into the analysis. The paper is a first step to broaden our understanding of inactive youth.

## Method

Data used in the present study were drawn from the research project the “Relevance of Physical Activity Contexts in the Everyday Life of Adolescents” (REPAC). The observational part of the project was based on a quantitative survey presented annually for two birth-cohorts of students (secondary and upper secondary school) during their 3 years at school.

### Participants and procedures

The present study used data from both cohorts collected at the end of their third and last year at secondary school (year 10; age 15–16) and upper secondary school (year 13; age 18–19) respectively. Students from 44 different schools in three different Norwegian counties participated, and this study comprises a total of 2575 participants. From this sample we identified 531 students who reported no involvement in sports or PA outside mandatory school settings and 2044 who reported outside-of-school involvement in organized sports, self-organized PA, or both.

### Ethics approval information

Data were collected in each of the participating schools during regular school hours using a web-based program for conducting electronic questionnaires. A project researcher who was present during the data collection was able to answer questions related to the survey. Students were informed about the project prior to the data collection, including that participation was voluntary and that they were free to withdraw from the study at any time without providing a reason. The data collection was approved by the Norwegian Center for Research Data (NSD), the ethical committee at the Norwegian School of Sport Sciences who had ethical responsibility for the project, and the principals at each of the participating schools. Ethical approval from the regional committees for medical and health research ethics was not required. With respect to participants who were younger than 15 years of age, parental consent was obtained. Details about the project have been presented elsewhere (Erdvik, 2020; Erdvik et al., 2020).

### Measures

#### Physical inactivity – activity

*Physical inactivity – activity* was assessed by the following question developed by Sagatun et al. (2007): “How many hours per week do you spend on physical activity (in addition to mandatory physical education in school) to an extent that make you sweat and/or out of breath”; 0, 1–2, 3–4, 5–7, 8–10, or 11 hours or more per week. To get a clean distinction between active and inactive youth, the data were assessed dichotomously. Inactive youth were coded 1 and active youth were coded 0.

#### Demographics

Information about gender, newcomer status (lived all years in Norway or not), secondary versus upper secondary school level, and self-reported academic grades (English/Norwegian/Math) were registered through single and straightforward questions.

#### Perceived socio-economic status

*Perceived socio-economic* status was assessed using the MacArthur scale of subjective social status (Adler et al., 2000). The students were asked to imagine a ladder with 10 rungs where highly educated families who are well off and have the most respected jobs are at the top and families with little or no education, unemployed or with jobs that are poorly paid are at the bottom. The students were then asked to range the position of their family compared to others in Norway.

#### PA identity

*Physical activity identity (PAI)* was evaluated using a four-item scale developed by Lorentzen and MoRo (2007). Participants responded to the four statements about their relationship to physical activity (e.g. “I view myself as a person who is concerned about physical activity”) with answer options ranging from (1) “suits badly” to (5) “suits well.” Cronbach’s alpha in the present sample was 0.94.

#### Eagerness for PA

*Eagerness for Physical Activity* was assessed using The Eagerness for Physical Activity Scale (EPAS; Säfvenbom et al., 2017). This one-dimension scale comprises nine items that evaluate both affective and cognitive aspects, such as an individual’s desire to engage in physical activity, their enjoyment, and the significance of physical activity to them. It also considers behavioral aspects, including the individual’s aspirations and plans to continue being physically active in the future. The items are presented as statements like “I always look forward to training or being physically active,” and “I am always happy when I have been practicing/been involved in physical activity” —with seven response options ranging from 1 (“Disagree completely”) to 7 (“Agree completely”). Cronbach’s alpha in the present sample was 0.96.

#### Social context

Two best friends’ behavior were assessed by a modified version of Friends’ Substance Use Questionnaire (Pape and Rossow, 2004). The respondents reported on six items that indicated their two best friends’ tobacco use, alcohol use, marijuana use, university plans, contacts with police, and involvement in sports or physical activity. Responses were measured dichotomously (yes/no) for friend 1 and friend 2. While items indicating positive behavior were coded 2, items indicating negative behavior were coded 1. Final scores are presented as mean scores for Friend 1 and Friend 2 respectively.

*Sense of school* was measured through three items assessing how the students perceived their school, their class, and the recess breaks (e.g. I enjoy being at school). Responses were measured on a seven-point Likert scale from (1) completely untrue to (7) completely true. Cronbach’s alpha for this measure was 0.89 in the present study.

#### Psychological factors

*Global self-worth, athletic competence, academic competence, and physical appearance* were assessed with the corresponding subscales from the revised Norwegian version (Wichstrøm, 1995) of Harter’s Self-Perception Profile for Adolescents (SPPA; Harter, 1988). The instrument assesses individuals’ self-perception generally (global self-worth) and domain-specifically. Each domain includes five statements (e.g. athletic competence: “I do well in all kinds of sports,” global self-worth: “I am very happy being the way I am”). Participants rated their agreement with these statements on a scale from (1) “Describes me very poorly” to (4) “Describes me very well.” In our sample, Cronbach’s alpha for global self-worth was 0.82, Cronbach’s alpha for academic competence is 0.72, Cronbach’s alpha for athletic competence was 0.78, and Cronbach’s alpha for physical appearance was 0.87.

*Life satisfaction* was assessed by the Satisfaction with Life Scale (SWLS; Diener et al., 1985). The Norwegian version has previously been shown to be appropriate for usage in an adolescent population (Moksnes et al., 2014) and it includes five items (e.g. “My life is satisfying”) that assess overall life satisfaction. Responses were measured on a seven-point Likert scale from (1) strongly disagree to (7) strongly agree. Cronbach’s alpha for this measure was 0.91 in the present sample.

*Psychological distress* was assessed by use of the Norwegian version of the 10-item Symptom Check List (SCL-10; Strand et al., 2003). This shortened form of the SCL-25 (Derogatis et al., 1974) includes four items related to anxiety and six items addressing depressed mood. Participants were asked to reflect on their experiences over the past week when responding to the items. Examples include “Feeling scared for no reason” (anxiety) and “Feeling of worthlessness” (depression). Responses were given on a four-point scale ranging from 1 (not at all) to 4 (extremely). Cronbach’s alpha was 0.91 for this measure in the present sample.

*Personal growth* was measured using the Personal Growth Composite as described by Straume and Vittersø (Straume and Vittersø, 2015). The instrument has been used as a global score of personal growth and includes a total of 12 items from 4 sub-components: curiosity, absorption, complexity, and competence. Participants rated their agreement with statements on a likert-type scale ranging from 1 (strongly disagree) to 5 (strongly agree). Examples of the items include: “I enjoy tackling complex problems,” “I often get so absorbed in activities that I lose track of time,” “I love coming up with new ideas,” and “I am capable of performing a wide range of tasks” for curiosity, absorption, complexity, and competence, respectively. Cronbach’s alpha for this measure was 0.90 in the present sample.

*Positive and negative emotions* were measured using the Basic Emotion State Scale (Vittersø et al., 2005). Using a Likert scale ranging from 1 (never) to 7 (always), the participants were asked to respond to what extent they during their everyday life experienced six positive emotions including hedonic emotions (satisfaction, pleasure, happiness) and eudaimonic emotions (interest, engagement, and immersion) and three negative emotions (anger, fear, and sadness) during their everyday life. All six positive emotions were merged to form a positive emotion mean score, and the items for anger, fear, and sadness were merged to create a negative state emotion mean score. Cronbach’s alpha for the negative emotion subscale in the present study was 0.83, and for the positive emotions subscale it was 0.88.

### Data analysis

We ran a classification and regression tree (CART) analysis (Krzywinski and Altman, 2017; Song and Lu, 2015) to examine how a wide variety of demographic, social, and psychological factors relate to physical inactivity. CART differs from logistic and linear regression approaches in that it does not result in an equation but rather a series of subgroups based on the relative importance of predictor (Krzywinski and Altman, 2017). This analytical approach is used in exploratory analyses in many different fields and is helpful for tasks such as identifying relevant variables to use in future research and evaluating the relative predictive value of variables within a model (Song and Lu, 2015). Youth development scholars are also beginning to adopt this approach to exploratory research (Anderson et al., 2023).

In the present study, we set the dichotomous variable of physical inactivity as the dependent variable, and included predictor variables related to demographics (gender, lived years in Norway, perceived SES, secondary vs upper secondary school level, grades in English/Norwegian/Math), social context (Friend 1 influence, Friend 2 influence), and psychological factors (satisfaction with life, HSCL, personal growth, positive emotions, negative emotions, general self-worth, athletic competence, academic competence, physical appearance, liking of school/belonging). We used the Chi-squared Automatic Interaction Detection (CHAID) function in SPSS (Version 29) with minimum parent node size set to 50 and minimum child node size set to 25. Missing data were included in the model as a meaningful value (Song and Lu, 2015). We also ran an initial set of variable-level ANOVAs and a correlation test including all study variables to examine potential differences between these analytic approaches and the CART method.

## Results

We first explored the direct relationships between inactivity and the demographic and psychosocial variables in our data set.

As shown in Table 1, multivariate analysis of variance MANOVA showed statistically significant differences (with a Bonferroni corrected *p*-value cutoff of 0.003) between active and inactive youth on 14/19 variables. The inactive group reported less promising scores on variables supporting quality of life and developmental processes, compared to their physically active peers.

**Table 1.** Multivariate analysis of variance MANOVA of differences between active and inactive youth.

Variables	Active youth	Inactive youth	*p*-value	eta-squared
School level (Sec. vs. upper sec)	1.66 (0.47)	1.52 (0.50)	<0.001*	0.015
Gender	1.49 (0.50)	1.48 (0.50)	0.827	<0.001
Newcomer status	1.09 (0.29)	1.13 (0.33)	0.049	0.002
Perceived SES	7.35 (1.64)	6.72 (1.87)	<0.001*	0.022
School grades	4.10 (0.83)	3.87 (0.85)	<0.001*	0.012
Friend 1 influence	1.80 (0.23)	1.74 (0.26)	<0.001*	0.013
Friend 2 influence	1.82 (0.21)	1.78 (0.21)	<0.001*	0.007
PA identity	5.40 (1.36)	3.79 (1.59)	<0.001*	0.177
Satisfaction with life	5.10 (1.28)	4.51 (1.42)	<0.001*	0.032
Psychological distress	1.70 (0.67)	1.79 (0.75)	0.009	0.003
Personal growth	3.39 (0.76)	3.11 (0.84)	<0.001*	0.02
General positive emotions	5.00 (1.11)	4.49 (1.22)	<0.001*	0.031
General negative emotions	3.03 (1.34)	3.16 (1.44)	0.056	0.001
Eagerness for PA	5.35 (1.30)	3.67 (1.56)	<0.001*	0.199
Global self-worth	2.97 (0.67)	2.81 (0.69)	<0.001*	0.009
Academic competence	2.98 (0.59)	2.76 (0.55)	<0.001*	0.022
Athletic competence	2.72 (0.67)	2.33 (0.64)	<0.001*	0.053
Physical appearance	2.70 (0.76)	2.61 (0.76)	0.018	0.002
Liking of school	5.76 (1.25)	5.37 (1.47)	<0.001*	0.015

* Indicates *p*-values below the 0.05 alpha cut-off after Bonferroni corrections for 19 tests.

Correlation analysis showed only gender and negative emotions were uncorrelated with inactivity, although gender was correlated with all other variables in the data set except liking of school, academic competence, and newcomer status, and negative emotions were correlated with all other variables except school level, school grades, and newcomer status. As shown in Table 2, inactivity was correlated with school level (−0.122**), newcomer status (0.046*), socioeconomic status (−0.150**), friend 1 influence (−0.115**), friend 2 influence (−0.082**), physical activity identity (−0.420**), satisfaction with life (−0.178**), HSCL score (0.052**), personal growth (−0.142**), positive emotions (−0.177**), eagerness for PA (−0.446**), self-worth (−0.095**), academic competence (−0.147**), athletic competence (−0.230**), physical appearance (−0.047**), liking of school (−0.121**), and school grades (−0.108**).

**Table 2.** Correlations among study variables.

Variables	Inactive	School level	Gender	Newcomer status	SES	School grades	Friend 1 influence	Friend 2 influence	PA identity	Satisfaction with life	Psych distress
Inactive	–										
School level	−0.122^**^	–									
Gender	−0.004	0.087^**^	–								
Newcomer status	0.046^*^	0.004	0.032	–							
Perceived SES	−0.150^**^	0.073^**^	0.083^**^	−0.056^*^	–						
School grades	−0.108^**^	−0.045^*^	−0.079^**^	−0.053^*^	0.157^**^	–					
Friend 1 influence	−0.115^**^	0.253^**^	−0.082^**^	0.010	0.021	0.043^*^	–				
Friend 2 influence	−0.082^**^	0.169^**^	−0.110^**^	−0.026	0.049^*^	0.058^**^	0.466^**^	–			
PA identity	−0.420^**^	0.054^**^	0.076^**^	0.000	0.249^**^	0.134^**^	0.159^**^	0.129^**^	–		
Satisfaction with life	−0.178^**^	0.094^**^	0.144^**^	−0.050^*^	0.314^**^	0.204^**^	0.190^**^	0.178^**^	0.417^**^	–	
Anxiety and depression	0.052^**^	−0.053^**^	−0.301^**^	−0.020	−0.165^**^	−0.051^*^	−0.158^**^	−0.128^**^	−0.199^**^	−0.523^**^	–
Personal growth	−0.142^**^	0.026	0.085^**^	0.048^*^	0.143^**^	0.198^**^	0.068^**^	0.080^**^	0.325^**^	0.385^**^	−0.151^**^
Positive emotions	−0.177^**^	0.060^**^	0.162^**^	−0.021	0.218^**^	0.230^**^	0.120^**^	0.102^**^	0.349^**^	0.589^**^	−0.399^**^
Negative emotions	0.038	0.015	−0.228^**^	0.003	−0.104^**^	−0.039	−0.121^**^	−0.108^**^	−0.109^**^	−0.351^**^	0.550^**^
Eagerness for PA	−0.446^**^	0.100^**^	0.133^**^	−0.013	0.248^**^	0.136^**^	0.155^**^	0.144^**^	0.831^**^	0.436^**^	−0.220^**^
Self-worth	−0.095^**^	0.036	0.257^**^	0.018	0.188^**^	0.063^**^	0.115^**^	0.084^**^	0.236^**^	0.615^**^	−0.578^**^
Academic competence	−0.147^**^	0.022	0.029	−0.023	0.233^**^	0.460^**^	0.147^**^	0.137^**^	0.248^**^	0.452^**^	−0.347^**^
Athletic competence	−0.230^**^	0.021	0.302^**^	0.037	0.215^**^	0.045^*^	0.042^*^	0.010	0.531^**^	0.358^**^	−0.301^**^
Physical appearance satisfaction	−0.047^*^	0.009	0.311^**^	0.025	0.148^**^	−0.027	0.075^**^	0.060^**^	0.167^**^	0.462^**^	−0.494^**^
Liking of school/belonging	−0.121^**^	0.033	−0.005	−0.040	0.182^**^	0.178^**^	0.193^**^	0.179^**^	0.293^**^	0.494^**^	−0.363^**^

	Personal growth	Positive emotions	Negative emotions	Eagerness for PA	Self-worth	Academic competence	Athletic competence	Physical appearance satisfaction	Liking of school/belonging
Inactive									
School level									
Gender									
Newcomer status									
Socioeconomic status									
School grades									
Friend 1 influence									
Friend 2 influence									
PA identity									
Satisfaction with life									
Psychological distress									
Personal growth	–								
Positive emotions	0.518^**^	–							
Negative emotions	−0.088^**^	−0.198^**^	–						
Eagerness for PA	0.399^**^	0.431^**^	−0.117^**^	–					
Self-worth	0.319^**^	0.504^**^	−0.496^**^	0.279^**^	–				
Academic competence	0.379^**^	0.423^**^	−0.311^**^	0.259^**^	0.502^**^	–			
Athletic competence	0.318^**^	0.315^**^	−0.220^**^	0.570^**^	0.379^**^	0.285^**^	–		
Physical appearance satisfaction	0.240^**^	0.373^**^	−0.413^**^	0.191^**^	0.795^**^	0.383^**^	0.383^**^	–	
Liking of school/belonging	0.251^**^	0.420^**^	−0.277^**^	0.266^**^	0.386^**^	0.357^**^	0.145^**^	0.260^**^	–

* Indicates *p*-values below the 0.05 alpha cut-off.

** Indicates *p*-values below 0.01.

### Classification and regression tree results

Because sample size can affect CART results (i.e. classification accuracy is computed based on the number of cases in the node) we drew a random sample of active youth (*N* = 531) to compare to the inactive youth (*N* = 531). After confirming that there were no statistically significant differences between the subsample and other active youth on any of the variables to be used in the decision tree, we began our CART analysis.

We first ran a CART analysis using only demographic factors (school level, gender, newcomer status, SES, and grades) as inputs, to set a baseline for model improvement. The risk estimate for this initial model was 0.403 (standard error = 0.015). The model was 59.7% accurate overall, with an accuracy of 49.5% for active youth and 69.9% for inactive youth. This CART analysis showed the inactivity of both secondary school and upper secondary school students being primarily associated with their school grades (i.e. academic performance). Higher grades were associated with a lower likelihood of being inactive, although secondary school students with high grades were more likely to be inactive if they were newcomers to Norway. Figure 1 shows these results.

**Figure 1.**
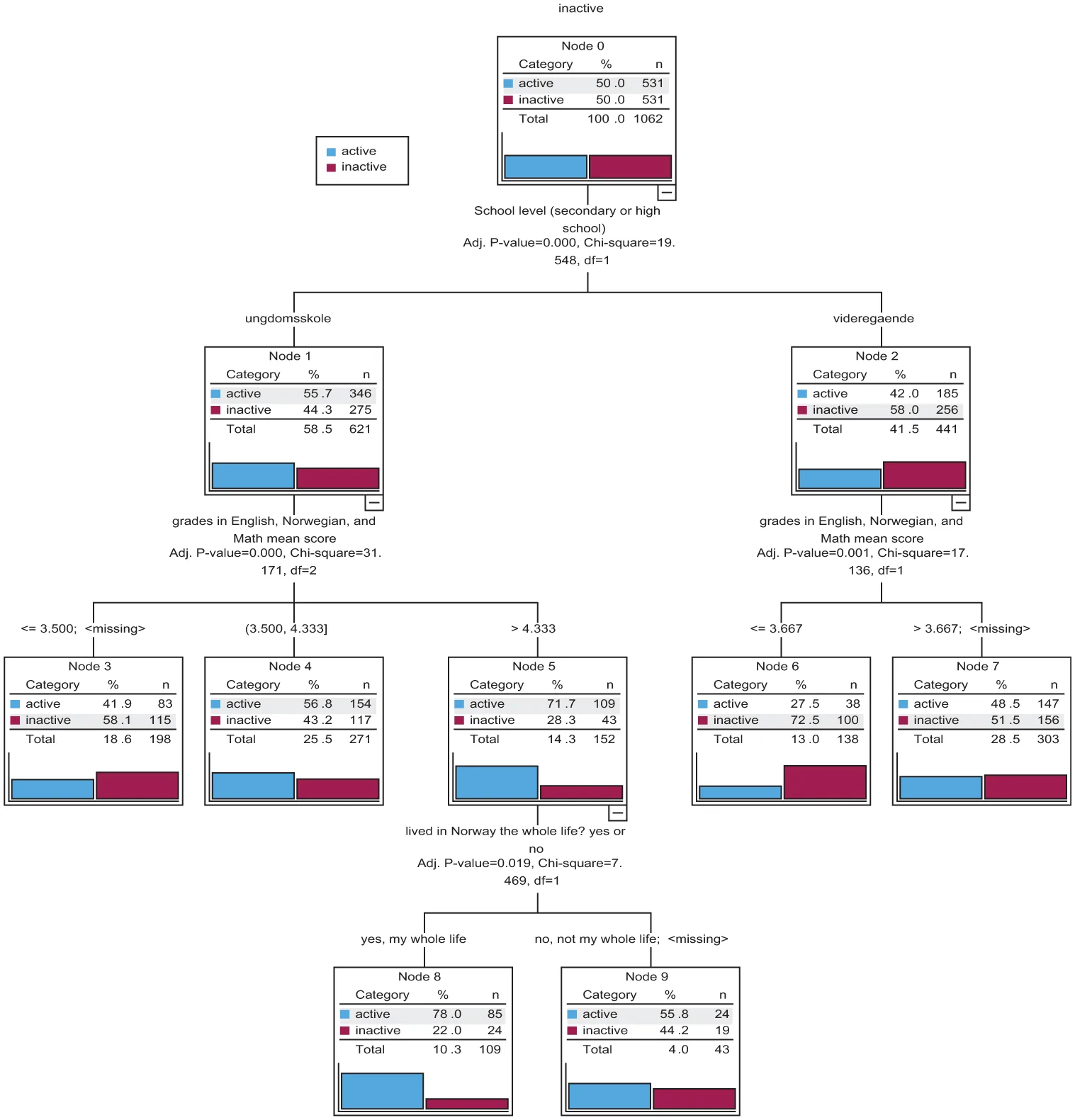
CART analysis results including only demographic factors.

We next ran a CART analysis adding psychosocial factors as inputs, and the risk estimate for this model improved to 0.241 (standard error = 0.013). The model was 75.9% accurate overall, with an accuracy of 71.2% for active youth and 80.6% for inactive youth. This CART analysis showed that youth inactivity was primarily associated with their level of eagerness for PA; the likelihood of being inactive was higher in nodes representing lower levels of eagerness for PA. The node representing the second lowest level of eagerness (eagerness = 2.11–3.0) was further split by school level, such that upper secondary school students with this level of eagerness were much more likely to be inactive. The node representing mid-high eagerness (eagerness = 4.0–5.11) was further split by physical activity identity, such that youth with this level of eagerness who had lower identity scores (less than or equal to 4.0) were more likely to be inactive. Furthermore, among the youth in this node with higher identity scores, those with higher levels of satisfaction with life (greater than 6) were more likely to be inactive. The node representing the second-highest level of eagerness (eagerness = 5.11–6.11) was further split by grades, such that youth with better grades in English, Norwegian, and math were less likely to be inactive. Finally, the node representing the highest level of eagerness (greater than or equal to 6.11) was further split by school level, with more inactive youth in the upper secondary school group, and with secondary school students being further split by physical activity identity such that lower identity scores (less than or equal to 5.75) were more likely to be inactive. Figure 2 shows these results.

**Figure 2.**
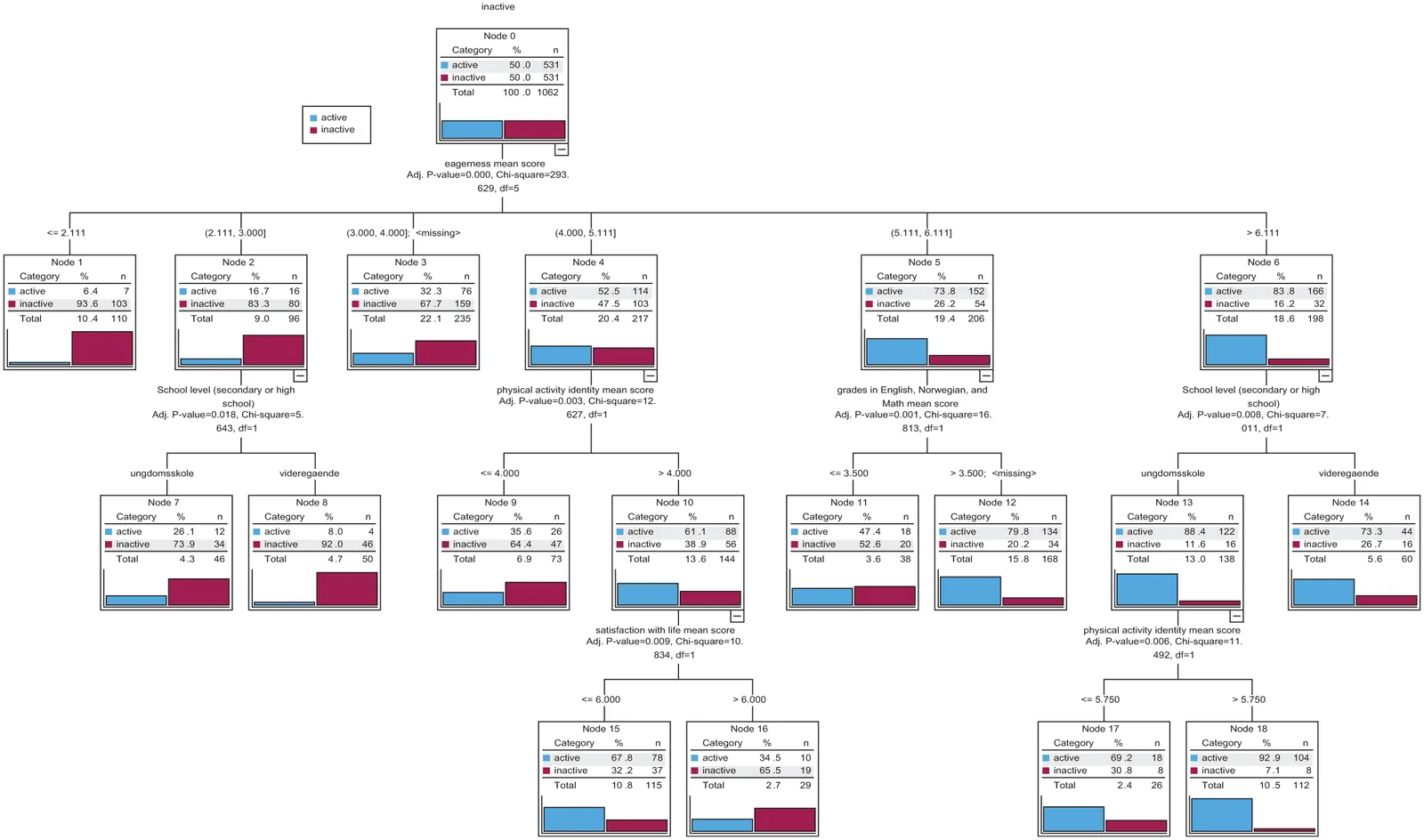
CART analysis results including both demographic and psychosocial factors.

## Discussion

Analyzes performed in this study show that associations between demographic variables and inactivity will inevitably emerge if psychosocial variables, representing corresponding relational and developmental systems, are not included. However, the psychosocial variables included in this study (see Table 1) represent both a primary distinction between active and inactive youth and interact with demographic factors. Our first CART model based on demographic variables showed a split by school level indicating more inactive youth in upper secondary school compared to secondary schools. This finding is consistent with prior research showing that older youth are less physically active than younger children (Steene-Johannessen et al., 2021). We also observed that inactive youth reported less good school grades at each grade level. Good grades usually reflect time and effort spent on schoolwork, and the results indicate that study hours per se do not necessarily reduce time for physical activity. These results are aligned with Swedish findings that PA is associated with higher levels of academic achievement (Kwak et al., 2009), but it is important to note that these correlational data do not speak to a causal mechanism. The final significant result in our initial CART model was that students who had lived all their years in Norway were more active than those who had not. This is very much in line with prior research on physical activity among newcomers in Norway (Enjolras et al., 2024).

When adding psychosocial variables to the model, the accuracy of the model improved. In the new integrated model, most of the nodes/branches were related to psychosocial variables, with a few secondary branches based on school level and grades. Compared to the demographic CART-model that was first split by school level, the integrated CART-model was first split by eagerness for physical activity. The fact that the concept of eagerness, and eagerness for physical activity in particular, is grown out of process-relational paradigms and holistic theories on human developmental (Säfvenbom et al., 2017), and that eagerness has been tested as a predictor for physical activity (Mikalsen et al., 2019), makes this result interesting also from an applied perspective. Prior research has shown that eagerness for physical activity is associated with perceived athletic competence and with moderate to vigorous physical activity (Mikalsen et al., 2019) and that change in eagerness for physical activity predicts change in VO2max (Säfvenbom et al., 2017). This makes eagerness for physical activity an interesting determinant, and it should be emphasized that compared to age, gender, social class, and other variables which cannot be affected by interventions, eagerness for physical activity is malleable. This means that we may find it more effective to develop interventions aiming not for more physical activity itself but for eagerness for physical activity. From a pedagogical point of view that is a significant difference.

All in all, our exploratory CART analyses show that demographic and psychosocial variables interact when they are both included to explain inactivity in youth, but that psychosocial variables seem to dominate. Furthermore, and highlighting the value of CART analysis, this approach enabled us to see that the interactions among psychosocial and demographic variables vary across different subgroups within the data set. Overall, our CART results correspond with, and expand on, the initial analyses performed where we compared our two groups’ response on 19 variables related to various developmental processes. The inactive group reported significantly lower scores on 14 variables including satisfaction with life, positive emotions, self-worth, athletic competence, academic competence, school grades, personal growth, and eagerness for physical activity. Based on the results presented, we suggest that psychosocial variables must be included in future analyses of active versus inactive young people. More research is needed, but when developing new interventions and activity programs for this group, the possibility that inactive groups of youth seem to live more challenging lives compared to their active peers and are generally less eager for physical activity must be considered.

### Limitations

This study is based on self-reported physical activity (Sagatun et al., 2007). We asked participants how often they were physically active to an extent that made them sweat and/or become out of breath. To classify individuals as either “physically active” or “inactive” (outside of physical education classes), we dichotomized the data. However, there are several sources of uncertainty related to the measurement used. First, self-reported physical activity is less precise than objective measures collected through devices such as accelerometers. Second, the expression “make you sweat and/or out of breath” may be interpreted differently by respondents. Third, dichotomizing the self-reported data into simply active and inactive reduces nuance in the activity levels reported. To conclude, we acknowledge that some respondents who in this study are classified as inactive, may engage in activities they do not associate with physical activity and who, with objective measurement, would likely be categorized as active.

Future research may benefit from examining the same psychosocial variables used in this study in relation to a continuous and objective measure of physical activity. National and international recommendations for daily physical activity may be used to distinguish between active and inactive youth. Follow-up studies might also include variables such as hours spent at school, part-time employment, and other leisure activities to better control for potential confounding variables. Finally, it should be noted that the sample of youth included in this study is not nationally representative. Participants were drawn from three counties in the south and southeast of Norway. While this may influence reported levels of physical activity, there is no reason to believe that the observed differences between active and inactive youth are affected by this limitation.

### Applications

Despite the limitations mentioned above, the understanding of inactive youth presented in this study makes the term “hard to reach group” even more suitable. The new understanding provided by our exploratory data makes it easier to understand why this group is hard to reach and why new and more holistic perspectives on physical inactivity are needed. Youth who experience themselves and their interactions with their surroundings in less positive ways may not see physical activity as a key solution to their challenges. Particularly when eagerness for physical activity is low and the reason for this most probably is to be found in former negative experiences from sports and physical education contexts (Erdvik et al., 2019a; Persson et al., 2020; Säfvenbom et al., 2014), physical activity is not given high priority. Inactive may also see PA in general, and mandatory PE in school in particular, as a threat to themselves and something they try to avoid. Because physical education may be the only PA context this group relates to, more research on their experiences with PE and mandatory physical activity programs in school is needed (Erdvik et al., 2019a).

Eagerness for physical activity requires positive movement experiences (Agans et al., 2013) because “human beings possess not only the ability to move, but also to explore, appreciate, value and develop their bodily interactions in terms of positive movement experiences” (Säfvenbom et al., 2017: 13). This means that reaching rather vulnerable and hard to reach groups requires process-oriented, exploratory, and experience-oriented approaches rather than pure mechanistic and instrumental epidemiological approaches. The term “Positive Movement Experiences” (PMEs) describes adaptive individual ← → context relations that can take place when individuals participate in movement contexts. Within this relational approach, it is suggested that if the characteristics of the individual and the characteristics of the context are aligned in such a way as to produce mutually beneficial relations, it is possible for all individuals to have PMEs (Agans et al., 2013: 213). If a young person is low on self-esteem, positive emotions, efficacy, or attribution and a physical activity program makes positive changes to these variables, physical activity may become associated with positive experiences (Agans, 2024; Agans et al., 2024). When physical activity or involvement in physical activity contexts becomes a means to experience something that protects the self rather than threatening the self, change in behavior may follow.

## Conclusions

The results presented in this paper highlight a need for more integrative analyses when looking at inactivity in youth, and a corresponding need for more holistic perspectives when we address inactive youth with relevant physical activity programs. Our results suggest that when we feed statistical analyses with only demographic data such as age, gender, social class, and ethnicity, we fail to examine the role of important psychosocial variables. When this happens, we miss beneficial knowledge about inactive youth and thus miss a pedagogical potential for developing appropriate interventions. The present study confirms that concepts and theories underpinning terms like Eagerness for Physical Activity and Positive Movement Experiences are important when developing interventions for “hard to reach” groups of youth.

## Data Availability

The data that support the findings of this study are available from the corresponding author, [R.S.], upon reasonable request.*
